# Advanced practice registered nurses: A solution for primary care shortages in sub‐Saharan Africa

**DOI:** 10.1002/nop2.1909

**Published:** 2023-06-14

**Authors:** Serina Gbaba, Jacqueline Itambo

**Affiliations:** ^1^ Johns Hopkins University School of Nursing Baltimore Maryland USA; ^2^ College of Nursing Colorado State University Pueblo Colorado USA

Sub‐Saharan Africa (SSA) has 15% of the world's population (1.18 billion), 66% burden of disease due to non‐communicable diseases and only 3% of the global healthcare workforce. (The World Bank Group, 2023). Nearly, all SSA countries have severe shortages of nurses and doctors with estimated ratios of 2.3 healthcare workers per 1000 population compared to high–income countries such as the Unites States of America (USA) with 24.8 healthcare workers per 1000 population (Miseda et al., 2017; The World Bank Group, 2023). With an estimated annual population growth rate of 3%–4% (World Bank Group, 2023), World Health Organization (WHO) estimates the SSA shortage for healthcare workers to reach 6.1 million by 2030 (WHO, 2023). Global disease burden dominated by acute and infectious diseases continues to be a significant burden in SSA with a reported 90% of malaria infections, 26 million people infected with human immunodeficiency virus and 10 million people infected with tuberculosis (Velavan et al., 2021). Like western countries, SSA countries have seen an increasing trend in chronic diseases such as ischaemic heart disease, stroke and diabetes for the last 25 years worsening the existing disease burden (Abdulkader et al., 2018).

Advanced practice registered nurses (APRNs) defined by the American Association of Colleges of Nursing (AACN), are nurses prepared at the master's or post‐master's level in a clinical role or patient population (AACN, 2021). Various countries have different titles and scope of practice for APRNs but have similar reasons for establishment: to address the shortage of advanced provider workforce, improve healthcare access and provide cost‐effective care (Christmals & Armstrong, 2020).

To meet delivery of essential health services and achieve universal health coverage, the WHO recommends healthcare workforce density of 4.45 health workers per 1000 people (WHO, 2023). Studies done in developed countries where the APRN role has been in existence for several years, show that APRNs provide care that is cost‐effective, patient‐centered, and of high quality compared to other practitioners (Christmals & Armstrong, 2020). APRNs have proven to be cost‐effective and have been associated with shrinking shortage of primary care providers, decreased use of emergency care services and high‐quality care comparable to that of physicians (Rugs et al., 2021). Countries in SSA are grossly underserved and low‐resourced and would therefore benefit from the advanced practice APRN role to alleviate the primary care provider shortfall. There is a need for improving the region's preventative health initiatives (maternal and infection control) and management of chronic diseases such as human immunosuppressive virus, diabetes mellitus and cardiovascular disease. In a continent that continues to struggle with frequent outbreaks of infectious diseases such as Ebola, APRNs can positively contribute to disease prevention and management. Supporting this statement is evidence from documented APRNs responses to the COVID‐19 pandemic in the USA. During the pandemic, APRNs remained in the front line of care to treat, complete procedures, educate, attend to vulnerable populations, promote mental health and innovate COVID‐19 management strategies (Diez‐Sampedro et al., 2020). Successful APRNs' role in the management of COVID‐19 proved that they are essential in preparedness for future pandemics.

The WHO strongly advocates for increased access to primary care to improve the quality of care, prevent diseases and increase healthy life expectancy in SSA (WHO, 2021). APRN education and practice has proven to be a strategic path to reducing health inequality and providing access to healthcare across the lifespan. It is estimated that SSA nurses make up about 47% and doctors 9% of the healthcare workforce with an average annual growth in nursing workforce of 40% (Ahmat et al., 2022). To keep pace with the rapid population growth, SSA countries should invest in the largest and fastest‐growing workforce, the nursing profession. Nurses form the foundation for the APRN role through their education and clinical experience. A small percentage of doctors in SSA serve in urban areas leaving nurses as rural care providers in many parts of SSA. Developing an APRN role will increase primary healthcare access, especially in rural areas where the need is more dire.

Establishing APRNs role in SSA to address primary care needs will not be without challenges, similar to those encountered in high–income countries where the APRN role was initially conceived. Some countries in SSA such as Botswana, Ghana, Malawi, Nigeria, South Africa and Tanzania have attempted to start advanced practice nurse education, but lack of legislation and formal curriculum to guide the development of successful APRN programmes have been cited as challenges (Christmals & Armstrong, 2020). SSA countries can develop strategies for overcoming these barriers by examining the sucessful establishment of the APRN role in other countries.

In the USA, initially, the APRN programmes faced a lack of government regulation, pushback from physician groups, faculty shortage and limited resources to support the APRN programmes. Since its inception in 1965 as a single programme, the APRN role now has multiple specialty tracks such as specialized nurse partitioners, clinical nurse specialists, nurse anaesthetists and certified nurse midwives serving the healthcare needs across diverse patient populations in the USA (AACN, 2021). This has been possible through the voice of nursing leadership advocating for nursing education, nursing role in healthcare and collaborating with law lawmakers and other stakeholders in healthcare delivery. There is a need for a unified voice of nursing leadership in SSA to advocate for APRN education and its role in primary care to address healthcare needs.

International advocacy from prominent nursing organizations like the International Council of Nurses (ICN) and American Nurses Association (ANA) as well as funding and support from international organizations such as the WHO, United States Agency for International Development (USAID) and prominent non‐profit organizations are vital to the success of APRN programmes in SSA. These organizations can engage governments and institutions of higher education in SSA to promote and support APRN education and training. The WHO acknowledges the primary care issues in SSA and the APRN education as a strategic goal to achieve SSA universal health coverage. Organizations like the ICN can provide guidance to nursing organizations in SSA on developing a legal framework to lobby and collaborate with law makers to advance the agenda for APRN education and regulation. This support is key because nursing leadership organizations in SSA require capacity building, coaching and guidance since the process can be challenging and highly political due to opposition from influential and powerful stakeholders (Christmals & Armstrong, 2020). Prominent international nursing organizations can provide technical expertise to influence and promote SSA national nursing organizations and help them secure international grants to fund the APRN primary care initiative.

Effective academic preparation is essential for the success of the APRN role in SSA. Nursing councils in SSA countries can collaborate with the AACN, an organization with expertise and experience in developing the essentials and core competencies for professional nursing education in the USA (AACN, 2021). This kind of collaboration will be key in setting APRN education standards, scope of practice and required competencies. Partnerships with nursing schools in the United States will be indispensable for technical assistance in building and implementing sustainable academic programmes using evidence‐based practices and teaching strategies. This can be best achieved through student exchange programmes and faculty collaboration in teaching, service and scholarship. This kind of partnership and collaboration was a success story for Family Nurse Practitioner (FNP) role established in Eswatini, a small country in SSA. The framework of the FNP role was adopted from the USA by the Eswatini nursing council and revised to be relevant to local healthcare needs (Dlamini et al., 2020). The technical group working with representatives from training institutions, academic institutions, supporting partners and practice groups developed a successful FNP curriculum that enrolled the first group of students in 2017 (Dlamini et al., 2020). This was a successful initiative highly supported by the WHO which makes a compelling case for this call for action in SSA (Dlamini et al., 2020). There is compelling evidence to support the need for primary care in SSA which makes a persuasive case for developing the primary care APRN role. Since its inception, advanced nurse education and practice have been hailed a success in health education, health promotion and primary care with countries such as the United Kingdom, Canada and Australia reporting tremendous gains in improved healthcare quality and access (AACN, 2021; Miseda et al., 2017). The success is a remarkable applause to the nursing organizations that led the way, working persistently with stakeholders in establishing APRN role.

This editorial serves as a call for nursing leadership not only in SSA but also around the world to create a global collaboration of nursing organizations, academic institutions, health organizations, governments and lawmakers to establish successful APRN education and training programmes in SSA. As seen in other countries, establishing APRN role is not an easy task, but success is possible with a consistent focus on the end goal which is to increase access to primary healthcare and improve healthcare outcomes. The establishment of APRNs across SSA will ultimately decrease primary care shortfall, improve prevention and management of chronic diseases, and ultimately lead to improved population health.
